# Recent global increase in multiple rapid intensification of tropical cyclones

**DOI:** 10.1038/s41598-023-43290-9

**Published:** 2023-09-24

**Authors:** N. D. Manikanta, Sudheer Joseph, C. V. Naidu

**Affiliations:** 1 Indian National Centre for Ocean Information Services, Ministry of Earth Sciences, Hyderabad, India; 2 Department of Meteorology and Oceanography, College of Science and Technology, Andhra University, Visakhapatnam, Andhra Pradesh India

**Keywords:** Natural hazards, Climate sciences

## Abstract

The rapid intensification (RI ≥ 30 knots/24-h) of tropical cyclones (TCs) makes TC forecasting difficult, resulting in severe damage to property and life. Forecasting can get even worse if TCs have experienced RI more than once (referred to as "multiple-RI TCs") in their lifetime. On a global scale, the relation between long-term trends of multiple-RI TC frequency and maximum potential intensity (MPI) changes is investigated in this study. During 1981–2020, the frequency of multiple-RI TCs significantly increased at a rate of 1.2 TCs/decade, which was primarily due to the upper phases of TC becoming conducive to RI as MPI increased. Our analysis shows that the frequency of multiple-RI TCs has increased by 82.43% in 2000–2020 compared to that in 1981–2000, whereas the frequency of single RI TCs has increased by only 1.63%. The rise in MPI elevates the initial intensity at which a TC undergoes maximum intensification rate, making post-Tropical Storm stages of TCs conducive to RI. As a result, TCs can undergo RI multiple times even following a weakening before the intensity approaches MPI.

## Introduction

Tropical Cyclones (TCs) are one of the most destructive weather systems around the globe, which can result in catastrophic damage to the socio-economic lives of coastal populations during their landfall^1,2^. In view of the increasingly devastating effects caused by TCs in recent decades, it is very important to understand the recent changes in TC activity. Despite recent advances in assessing variations in TC intensity and associated potential destructiveness^3,4^, predicting TC intensity and intensification rate remains challenging^5–8^. Although TCs exhibit a range of intensification rates over the course of their lifetimes, in particular, the forecasting of Rapid Intensification (RI), defined as an intensity rise of 30 knots or more in a 24-h period^7^, has proven to be a challenging task for TC forecasters. Hence, most of the studies have focused on the identification of necessary conditions for RI, especially the role of upper ocean processes^9–11^, inner-core dynamics^12–14^, and large-scale environmental interactions^15,16^. The RI phase occurs in regions with warmer sea surface temperature (SST), low vertical wind shear, high lower troposphere moisture, and large-scale upper-level forcing from troughs or cold low^7^. As the formation, intensification, and RI of TCs involve common environmental conditions, there is no particular set of processes or mechanisms that can be treated as special for RI that are different from the non-RI TCs^17^. Thus, failure to forecast the RI of a TC can be attributed to our limited knowledge of TC intensification rates in response to varying environmental conditions^7^. The rate at which the TCs grow has a profound impact on their Lifetime Maximum Intensity (LMI) climatology^18^. In most ocean basins, the mean intensification rate of TCs is showing an upward trend^19,20^. In this case, examining variations in TC intensification rates across time and at different stages of TC evolution will help us to understand properly when RI events occur.

Previous studies on the variations in TC intensity over time indicate that TCs go through two stages of intensification before attaining LMI^21^. TCs grow at a slower pace during the initial development for tens of hours until some TCs progress to high intensities, where they grow at a rapid rate^22,23^. The dependency of intensity change on the initial intensity of TC was reported by many studies^24–28^. The intensity change of TC depends on the diabatic heating in the eye wall. The efficiency of warming in the eye wall is determined by the inertial stability inside the inner-core, which increases as TC intensifies^29^. As the maximum heating corresponds to the maximum intensity change, the onset of RI in the TC life cycle is similarly determined by its initial intensity^27,30,31^.

When a TC's intensity is substantially lower than its maximum potential intensity (MPI), the likelihood of RI is high^7^. As a result, the likelihood of TCs undergoing RI in their early stages, i.e., Tropical Depression (TD), Tropical Storm (TS) stages of life cycle is significant as compared to the post-TS stages (cat-1,2,3,4,5)^24,32^. In an analysis of global TCs during 1998–2008, it was found that the majority of RI events occurred during the TS stage, with only a few RI cases observed in post-TS stages^33^. The impact of climate change on RI characteristics, such as increased annual frequency and mean magnitude of RI^34,35^, changes in geographical distribution^36^, and seasonal fluctuations^37,38^ of RI, was reported. However, the distribution of RI events across the various stages of the TC life cycle, as well as recent changes, must be examined to improve our understanding of RI incidence within the TC lifetime. Furthermore, TCs can be subjected to RI more than once in their lifetime, which can be referred to as multiple-RI TCs. These multiple-RI TCs may enhance forecasting errors even further. There has been little research into the recent trends of multiple-RI TCs. The dependency of multiple-RI events on the initial intensity of TC has been reported, with the favourable initial intensity for the first RI being 35 knots and the Re-RI events being 45 knots and 75 knots^37^. The present study focuses on the variability in the evolution of intensification rates during the life cycle of TC, with a particular emphasis on the distribution of RI. We believe that this study will contribute to improving our understanding and ability to predict the RI in the life cycle of TC.

## Results

### Expansion of RI favorability to post-TS stages of TC

**Figure 1:**
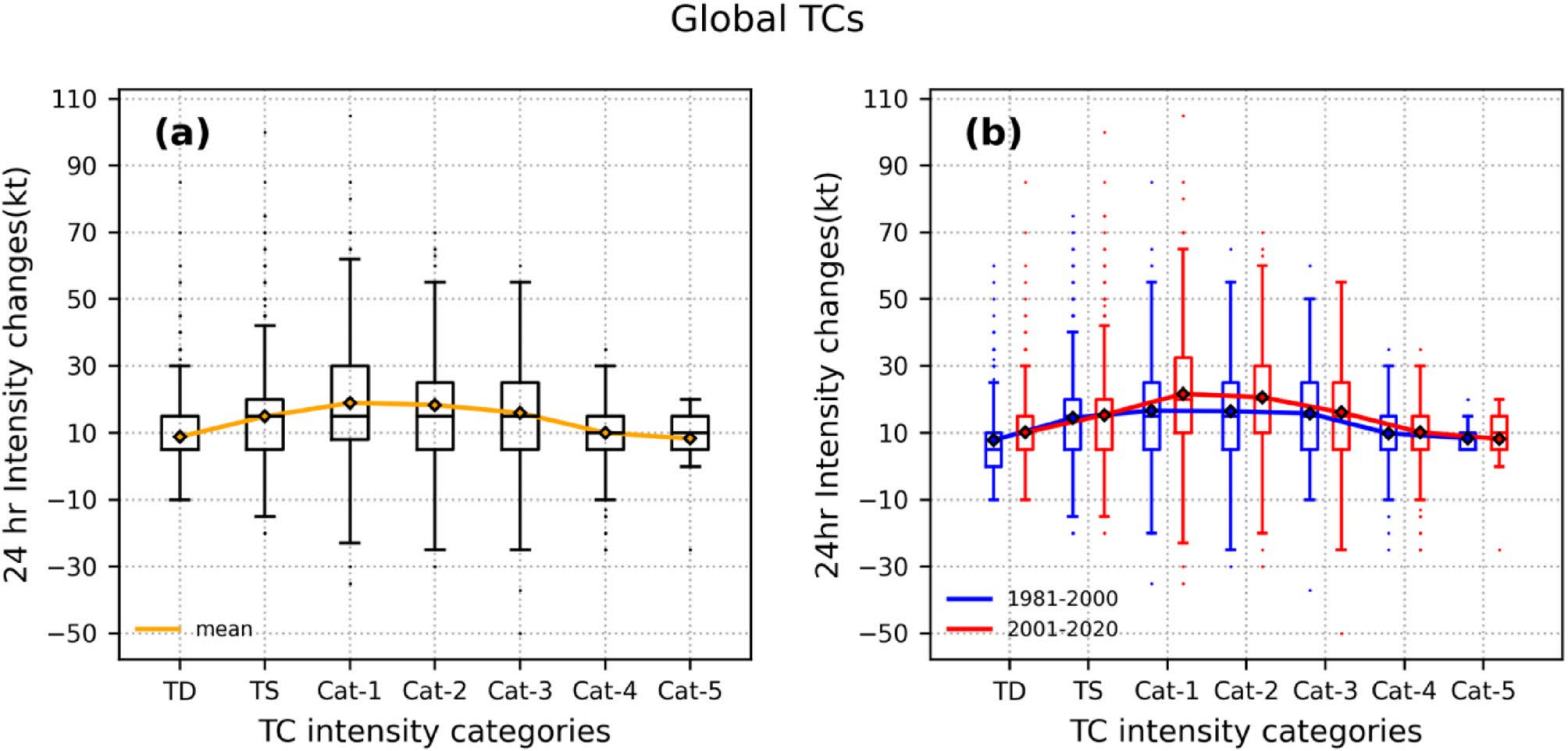
(**a**) Box plots of 24-h intensity changes (ΔV_24_) in different stages of the TC life cycle (intensification phase) that formed across the globe during 1981–2020. The Orange curve indicates the mean ΔV_24_ in each stage. (**b**) Shows the same, but for the two periods. Blue box plots show distribution during P1, and red box plots for P2, while the blue curve and red curve represent the mean values of intensity changes for the periods P1, and P2 respectively.

Figure 1a shows the distribution of 24-h intensity changes (ΔV_24_) in different stages of the intensification phase of TCs that formed around the globe during 1981–2020 and the orange curve in Fig. 1a represents the mean ΔV_24_ in each stage and (b) the changes in their distribution in the two periods 1981–2000 (P1); 2001–2020 (P2), where blue (red) box plots, blue (red) curves indicate the distribution and mean of ΔV_24_ in P1 (P2) respectively. We can see the distribution as well as the mean of ΔV_24_ clearly shows the dependency on storm intensity (category of TC). The mean ΔV_24_ increases with the category of TC until the storm reaches the cat-1 stage (V_max_ = 64–82 kt) and decreases during later stages of cat -1 in the intensification phase of TC. This is consistent with the results of earlier studies^27^, where it was shown that Intensification Rate (IR) of TC increases (decreases) with storm intensity when V_max_ is below (above) 70–80 knots (that falls into cat-1). For TCs at a relatively low intensity, the heating efficiency increases with intensity, which further intensifies the TC vortex because of the higher inertial stability of the inner-core^39^. As the storm crosses the cat-1 stage and approaches MPI, the increasing energy dissipation due to surface friction mostly counterbalances the higher heating efficiency energy^27,39^. The differences in the box plots in Fig. 1b represent changes in the distribution of ΔV_24_ in different stages of the TCs in the periods P1; P2. The major change was observed in cat-1,2 stages, with an increase in the respective mean values of ΔV_24_ by 5.029 kt, 4.149 kt respectively. A slight increase of 2.28 kt in the mean ΔV_24_ in the TD stage was also noticed. The ANOVA test results showed that the differences in the mean intensity changes among TD, categories 1 and 2 are statistically significant among the two periods.

**Figure 2:**
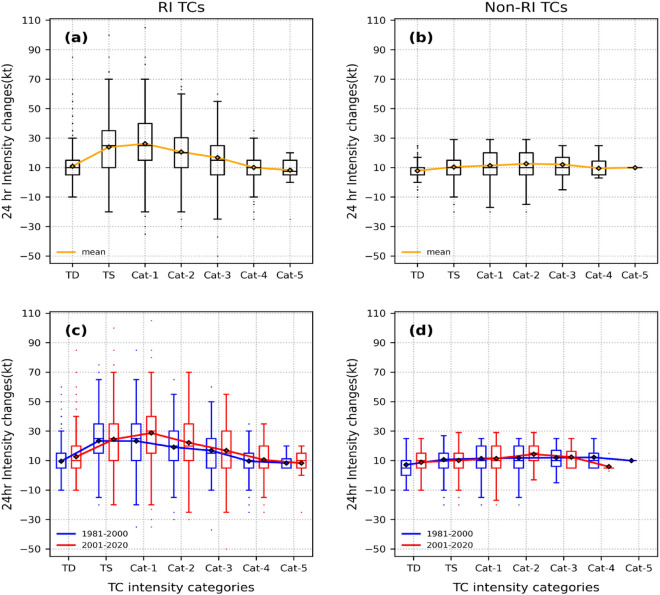
Box plots of 24-h intensity changes (ΔV_24_) in different stages of the TC life cycle (intensification phase) that formed across the globe during 1981–2020 for (**a**) RI-TCs ; (**b**) non-RI TCs. The orange curve indicates the mean ΔV_24_ in each stage. Box plots of 24-h intensity changes (ΔV_24_) in the two periods P1, P2 for (**c**) RI-TCs and (**d**) non-RI TCs. Blue box plots show distribution during P1, and red box plots for P2, while the blue curve and red curve represent the mean values of intensity changes for the periods P1, and P2, respectively.

The number of TCs undergoing RI has grown in recent decades^34^. The observed changes in Fig. 1b could be attributed to the increased frequency and magnitude of RI occurrences. To verify this, we computed the 24-h intensity changes for RI-TCs and non-RI TCs individually. The ΔV_24_ distribution in different stages of RI-TCs (Fig. 2a) differs from that of non-RI TCs (Fig. 2b). RI-TCs tend to intensify rapidly at the beginning of the TC life cycle, well before the TC intensity reaches close to MPI^7^. Non-RI TCs, on the other hand, intensify at a nearly uniform and low intensification rate throughout the intensification phase. As a result, only a small number of TCs that grow at non-RI rates make it to the major category (cat—3 and above). Figure 2c,d show the changes in the distribution of ΔV_24_ for RI-TCs and non-RI TCs respectively, for the two periods we considered in this work. The lack of significant changes in the ΔV_24_ distribution of non-RI TCs across the two periods (see Table S1) under consideration suggests that RI-TCs are primarily responsible for the changes in the ΔV_24_ distribution of total TCs (Fig. 1b). Previous research on the frequency distribution of RI events in different stages of TCs revealed that the TS stage had the highest proportion of RI events^33^. In agreement with this, the ΔV_24_ mean curve peaks in the TS stage during P1, however this peak has relocated to the cat-1 stage during P2. Together with this, the rising mean value of ΔV_24_ seen in the TD, cat-2 stage causes a shift in the distribution of ΔV_24_ in TD, cat-1 and cat-2 stages when all TCs are considered (Fig. 1b).

**Figure 3:**
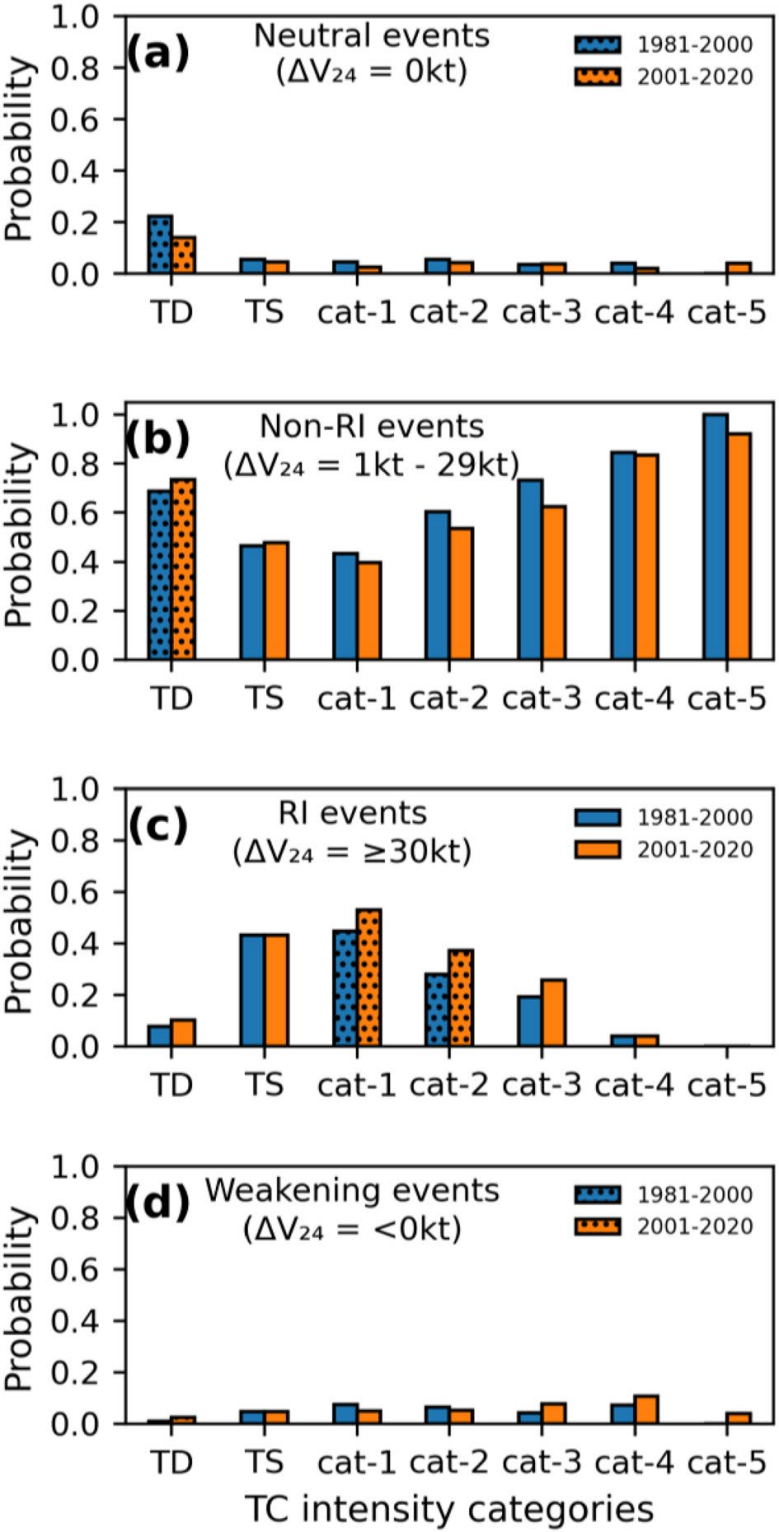
Probability distribution of different intensity change groups (**a**) neutral IR (ΔV_24_ = 0 kt) ; (**b**) non-rapid IR (ΔV_24_ = 1—29 kt); (**c**) rapid IR (ΔV_24_ ≥ 30 kt); (**d**) weakening rate (ΔV_24_ ≤ 0 kt) within each stage of the TC lifecycle (significant changes are displayed with hashed bars ).

As evident from the analysis so far, RI-TCs are responsible for the changes in the distribution of ΔV_24_ among different stages of the TC lifecycle. Hence, we consider only RI-TCs for further analysis. This section addressed the various intensity changes that may occur during a certain stage of TC, as well as how variations in those intensity changes affect the distribution of ΔV_24_. As mentioned in the methods section, we categorized the 24-h intensity variations into rapid, non-rapid, neutral, and weakening rates. For the two periods P1 and P2, Fig. 3 displays the variations in the probability of TC intensifying under the aforementioned classes within each stage. The cat-1,2,3 stages showed major changes. In the cat-1,2,3 phases, the likelihood of TC undergoing RI (non-RI) has increased (decreased) during the previous 20 years (Fig. 3b,c). This demonstrates that the increased probabilities of TC undergoing RI in the cat-1,2,3 are the cause of changes in the distribution of ΔV_24_ during the intensification phase. The probability of TC maintaining constant intensity in 24-h (neutral IR) is high in the TD stage, which is decreasing in the latter half of the study period (Fig. 3a). Furthermore, there is an increased likelihood of non-rapid and rapid IR in the TD stage during P2, indicating that TCs are increasing at a considerably faster rate so early in their life cycle, i.e., the TD stage. This early intensification shortens the time scale of TC life cycle^40^. Under favourable environmental conditions, TCs can intensify continuously until they approach the MPI. As a result, unless adverse environmental conditions exist, the likelihood of TC weakening during the intensification phase is relatively minimal. So, the weakening rate in the intensification phase has a very low probability, as evident from Fig. 3d. The probability of TC weakening has slightly increased in the higher categories cat-3,4,5 of the intensification phase because these stages are close to their respective MPIs (Fig. 3d).

**Figure 4:**
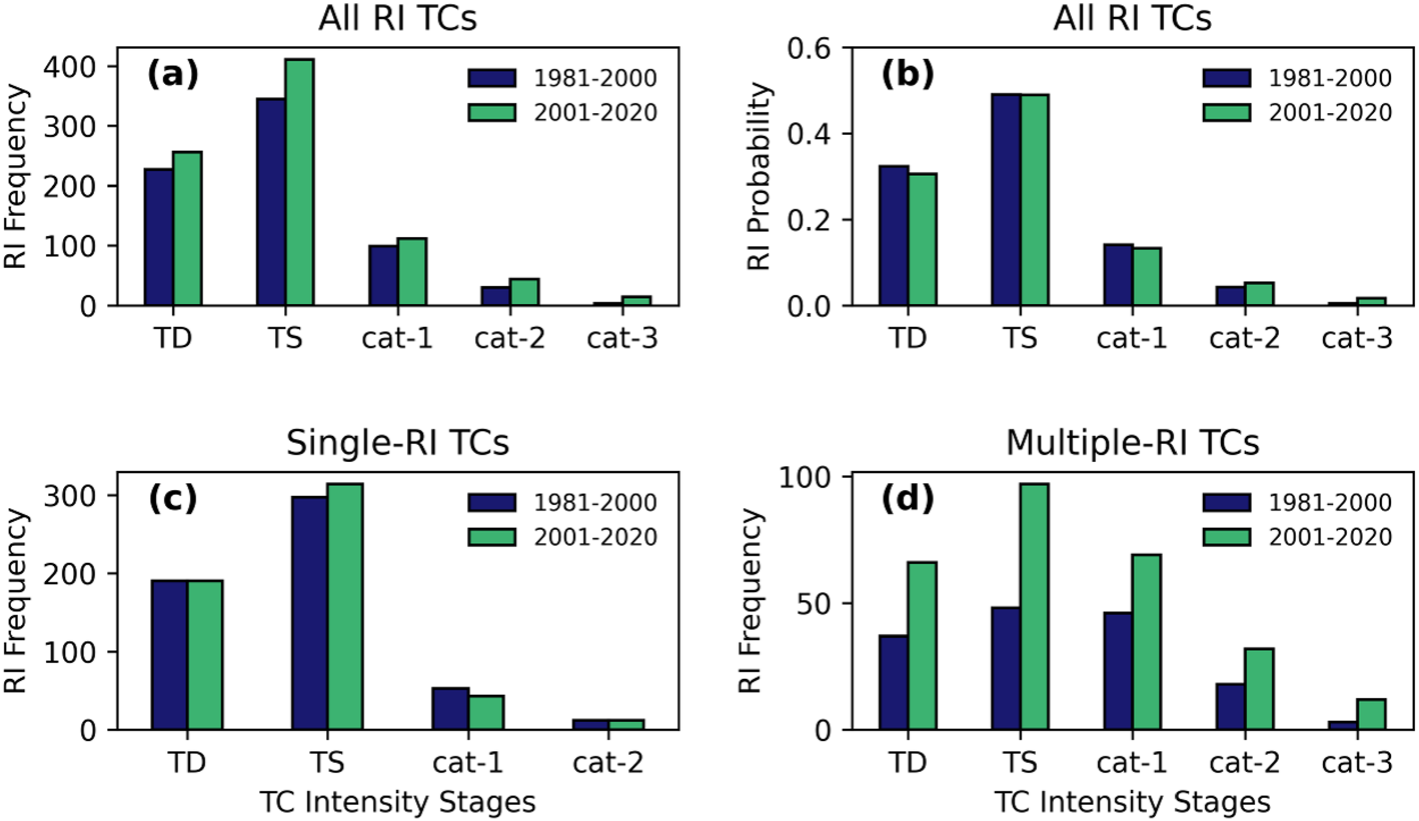
(**a**) Frequency distribution of RI events as a function of TC intensity categories for the period, P1 (dark blue); P2 (green) (**b**) Probability distribution of RI events among different stages of TC for the period, P1 (blue); P2 (green). Frequency distribution of RI for (**c**) single-RI TCs and (**d**) multiple-RI TCs.

**Figure 5:**
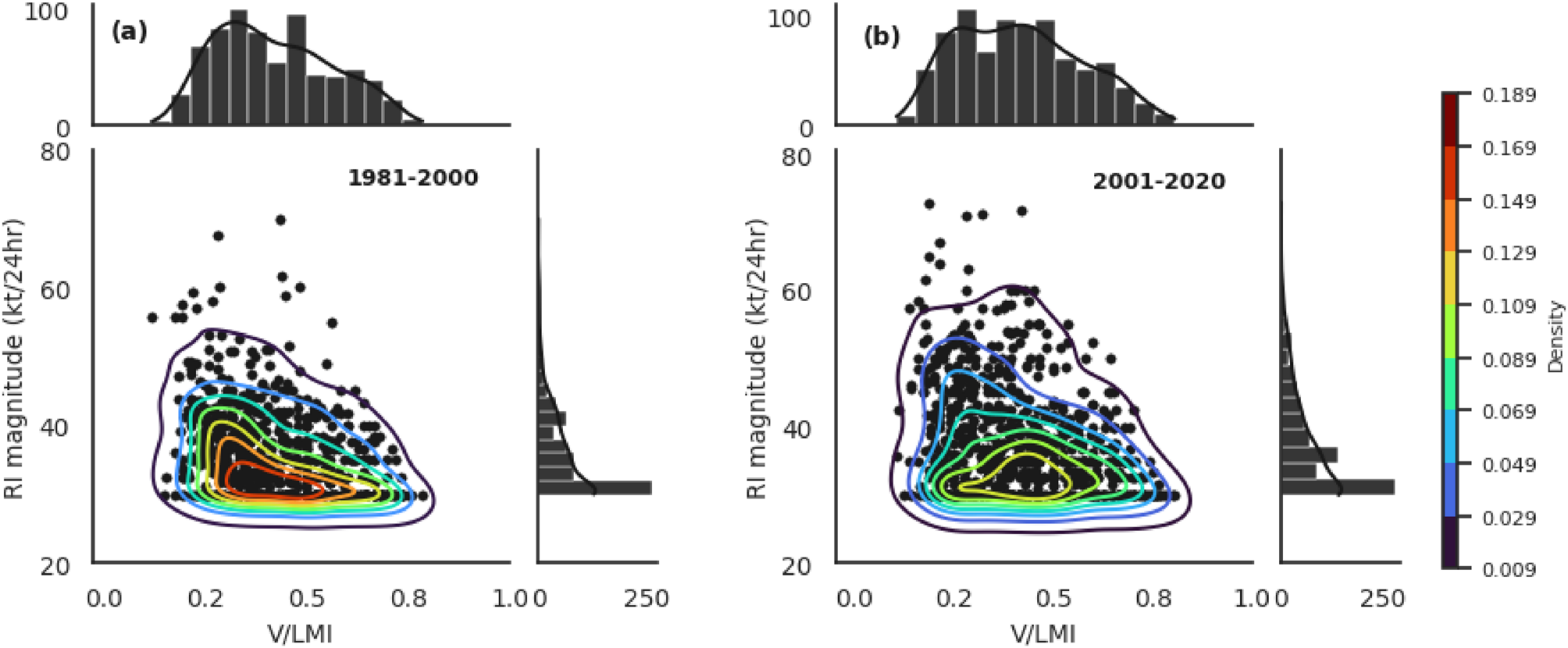
Density plots of RI magnitude against the initial storm intensity (V) during the RI event normalized by the lifetime max intensity (LMI) of the storm (namely V/LMI) for (**a**) 1981–2000; (**b**) 2001–2020.

Previous research study has shown that the TS stage is most advantageous for the RI of TC^33^. However, the growing likelihood of RI rates in cat-1,2,3 poses the question: Is the favorability of TC RI expanding towards the post-TS stages of TCs? Fig. 4a shows the variations in the frequency distribution of RI events at different stages of TC intensification. Consistent with earlier studies^7,33^, most RI cases have occurred in the TS stage of TC in both periods. However, a considerable increase in the frequency of RI cases was observed in all stages of TC from TD to cat-3 in our study, which has not been previously reported. Figure 4b shows the changes in the probability of TC undergoing RI among different stages. In both periods, the probability of TC undergoing RI is high in the TS stage, but in the recent past 20 years, this probability has grown in the cat-,2,3 stages (Fig. 4b). We also calculated the stage-wise distribution of RI occurrences in TCs having RI only once (single-RI TCs) and in TCs having RI more than once (multiple-RI TCs) separately. The onset of single-RI events is more common when the TS stage has progressed (Fig. 4c). In the instance of multiple-RI TCs (Fig. 4d), it can be seen that stages after TS have also supported RI occurrences, and their frequency is increasing. Figure 5a,b display the density of RI occurrences for various initial storm intensities (V) normalized by the storm's lifetime maximum intensity (LMI) during both periods (i.e., V/LMI). The peak density of RI events is seen in the first period of interest at 0.3, however, in the later period, additional peak density is visible at 0.5 in addition to the peak density at 0.3. The above results clearly prove that the favorability of TC to undergo RI has expanded towards post-TS stages of TC (Cat-1,2,3).

**Figure 6:**
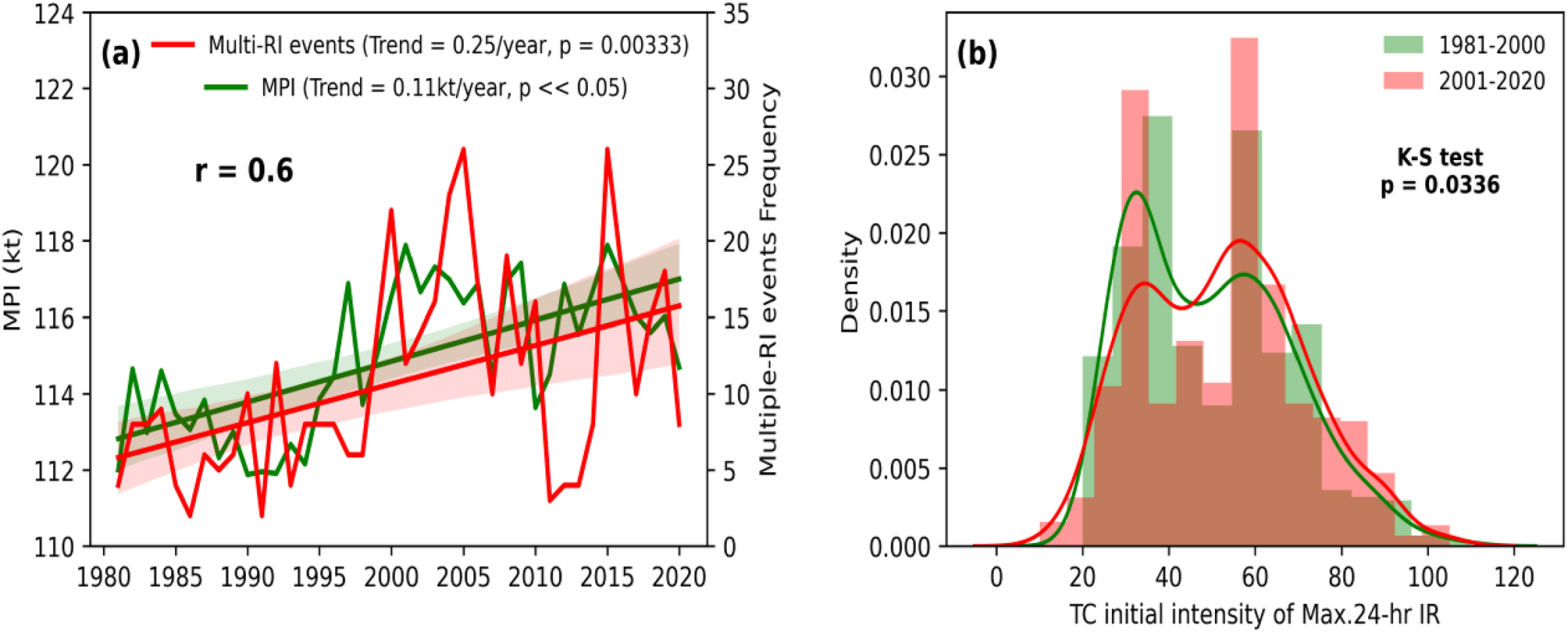
(**a**) Time series of Maximum Potential Intensity (MPI) (shown in green color) calculated for global tropical oceans (30N—30S) for the period 1981–2020 using ERA5 data along with time series of multiple-RI events (red) (**b**) Distribution plots of initial TC intensity of maximum intensification rate for RI TCs for the periods P1 (green) and P2 (red).

The intensification rate of TC in a particular stage depends on its initial intensity and how far that intensity differs from MPI^27^. Based on a simplified dynamical system for TC intensity prediction^31^, they have also shown that the intensification rate reaches a maximum when the TC is at its intermediate intensity relative to its LMI. Since the MPI is the theoretical limit of the maximum strength (LMI) that a TC can attain for a given set of favorable oceanic and atmospheric thermodynamic conditions, the initial intensity at which the maximum intensification occurs varies as the MPI changes. Under the global warming scenario, TC MPI is expected to increase with rising sea surface temperatures^41–44^. With increasing MPI (Fig. 6a), initial intensity, where TC shows maximum intensification rate, shifts to higher ranges (Fig. 6b). So during P2, the probability of TC intensifying rapidly in the cat-1,2,3 phases of TC increased, resulting in an increase in multiple-RI occurrences. As a result, the MPI is positively linked (r = 0.6) with annual multiple-RI events.

### Increasing multiple-RI events as a consequence of frequent RI at post-TS stages.

**Figure 7:**
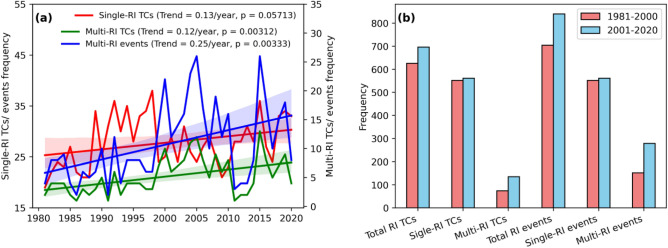
(**a**) Time series and trend of annual single-RI TCs frequency (red) and multiple-RI TCs frequency (green) and multiple-RI events frequency (blue) during the period 1981–2020. (**b**) Frequency changes of total RI-TCs and total RI events, single-RI TCs and events, multiple-RI TCs and events between the periods P1 and P2.

This section, looks at the impacts of increasing RI in post-TS stages on multiple-RI events. Figure 7a shows that the annual frequency of TCs that underwent RI only once (single-RI TCs) is rising at a rate of 1.3 TC per decade (p = 0.05). The frequency of TCs with RI more than once (multiple-RI TCs) is increasing at a rate of 1.2 TC per decade (p = 0.003), which is similar to the trend of single-RI TCs. Though single-RI and multiple-RI TCs are growing, the number of multiple-RI events is growing at about twice the rate. Figure 7b depicts the changes in RI frequency between the two periods of interest. During P2, there is an apparent increase in the total number of RI-TCs and RI events (Fig. 7b). Multiple-RI TCs (events) have increased by 82.43% (83.55%), but single-RI TCs have increased by just 1.63%. We verified the analysis using data from WMO agencies in the IBTrACS database to ensure robustness for P2 (see Fig. S5). This approach confirms the increasing trends in multiple-RI of TCs. In the case of a low MPI, TC can only experience RI during their early phases (TD, TS). Once the TC intensity crosses these stages, it approaches the theoretical upper limit of TC intensity (MPI), which is not favourable for RI. Because of the increasing MPI, the post-TS stages of TC are more suitable for RI, as they are still far enough from the theoretical upper limit of TC intensity. The early intensification of TC, paired with post-TS stages favouring RI, has raised the possibility of multiple RI episodes, increasing the frequency of multiple-RI TC.

**Figure 8:**
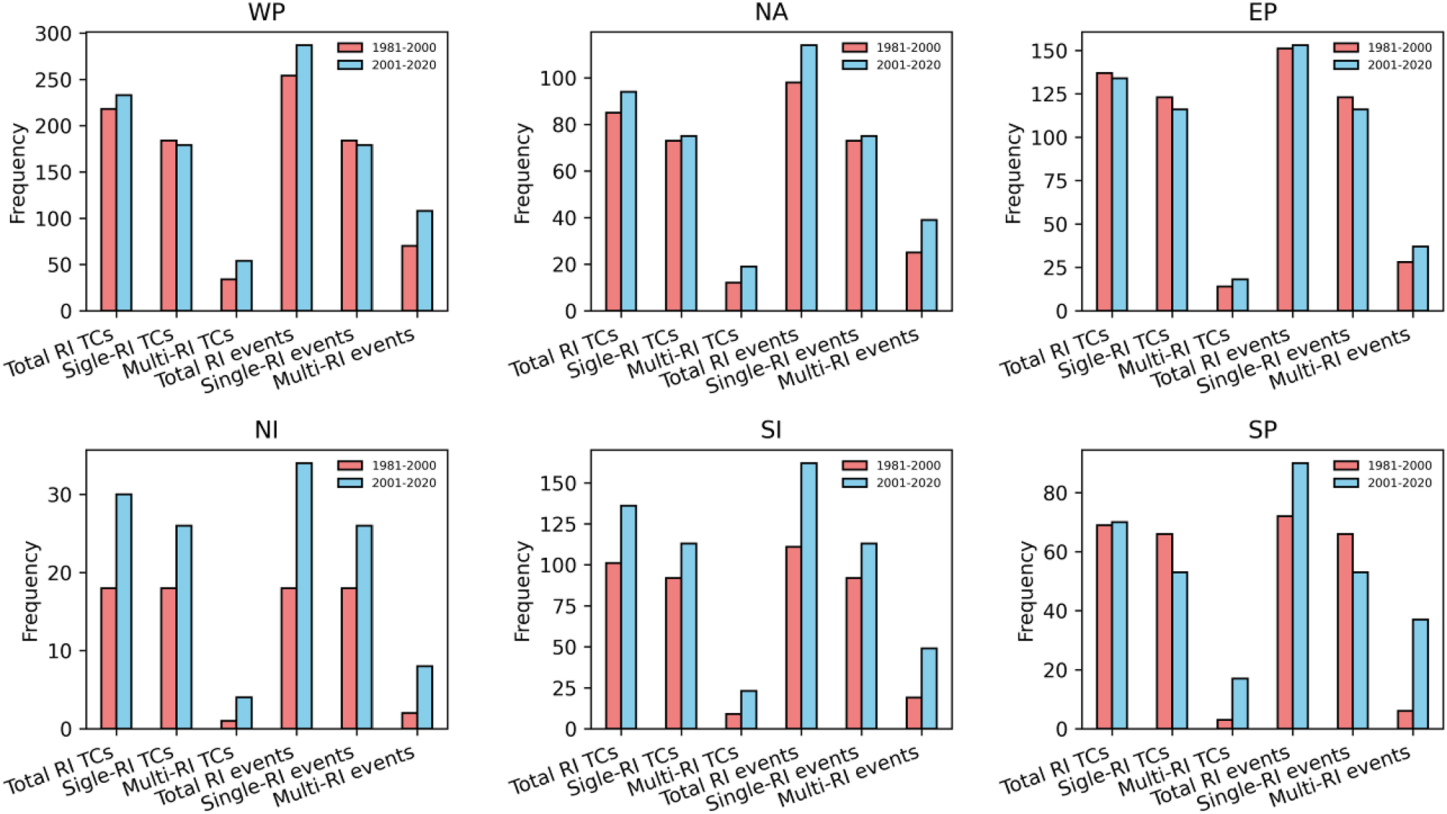
Basin wise frequency changes of total RI-TCs and total RI events, single-RI TCs and events, multiple-RI TCs and events between the periods P1 and P2.

We have investigated the situation of these multiple-RI trends in different ocean basins (see Table 1 and Fig. 8). Remarkably, a significant surge in frequency was observed across all ocean basins for both multiple-RI TCs and multiple-RI events. The Western North Pacific Ocean basin stands out with a substantial contribution, accounting for nearly half (42.1%) of the total occurrences of multiple-RI TCs. Following closely, the Eastern North Pacific and South Indian Ocean basins each contribute 15.3% to the global count of multiple-RI TCs. Moreover, the North Atlantic and South Pacific Ocean basins contribute to global multiple-RI TCs, accounting for 14.8% and 10% of the occurrences, respectively. The North Indian Ocean basin has a comparatively lower representation, contributing 2.3% to the overall count of multiple-RI TCs. However, the specific contributions of TC activity in different Ocean basins to the total rise in global multiple-RI TCs are diverse. With a considerable contribution of 32.78%, the Western North Pacific Ocean basin takes the lead.

**Table 1:** Frequency change percentage of the multiple-RI TCs and events (in brackets) between the two study periods.

Ocean basins	Multiple-RI TCs (events) change within basin	Contribution to Global Multiple-RI TCs (events)	Contribution to changes in Multiple-RI TCs (events)	Multiple-RI TCs Trend (p value)	Multiple-RI Events Trend (p value)
Golbal	82.43% (83.55%)			0.121/yr (p = 0.003)	0.253/yr (p = 0.003)
WP	58.82% (54.28%)	42.1% (41.3%)	32.78% (29.92%)	0.03/yr (p = 0.22)	0.05/yr (p = 0.26)
NA	58.33% (56%)	14.8% (14.8%)	11.47% (11.02%)	0.027/yr (p = 0.03)	0.053/yr (p = 0.05)
EP	28.57% (32.14%)	15.31% (15.08%)	6.55% (7.08%)	0.01/yr (p = 0.6)	0.02/yr (p = 0.5)
NI	300% (300%)	2.4% (2.29%)	4.91% (4.72%)	0.01/yr (p = 0.15)	0.02/yr (p = 0.15)
SI	155.55% (157.89%)	15.31% (15.7%)	22.95% (23.62%)	0.03/yr (p = 0.04)	0.06/yr (p = 0.04)
SP	325% (375%)	10.04% (10.7%)	21.31% (23.62%)	0.03/yr (p = 0.01)	0.065/yr (p = 0.009)

Four columns from right indicates Changes in frequency within each basin, as well as basin-specific contributions to global multiple-RI TCs, and their share in recent changes respectively. Basin wise trends of multiple-RI TCs and events, their p value in brackets are shown in last two columns.

Similarly, multiple-RI TCs increased in the South Indian and South Pacific Ocean basins during the last two decades, contributing 22.95% and 21.31%, respectively to total increase of multiple-RI TCs. In the North Atlantic, hurricanes also exhibited a notable surge in multiple-RIs, contributing 11.47% to the overall increase of multiple-RI TCs. While the Eastern North Pacific basin has a substantial proportion of multiple-RI TCs, its contribution to the recent surge is only 6.55%. Similarly, the North Indian Ocean basin contributes the least to the recent changes in multiple-RI TCs due to its relatively lower TC activity. We have examined the changes in environmental fields (SST, MPI, vertical wind shear and relative humidity) between the years 1981–2000 and 2021–2020 (Fig. S6). The increase in multiple-RI TCs can be primarily attributed to rising SSTs (Fig. S6a), particularly the MPI (Fig. S6b), in the Western North Pacific, South Indian Ocean, South Pacific, and North Atlantic ocean basins. At the same time the Eastern North Pacific and North Indian Ocean's smaller contributions to the multiple-RI TCs rise can also be attributed to decreasing MPI in sub-regions of the respective basins (see Fig. S6b). Although the Central and Northeastern Pacific have seen a noticeable increase in vertical wind shear (as shown in Fig. S6c), minimum changes has been seen in the majority of the the TC-active regions. In addition, there is a noticeable increase in relative humidity in the western North Pacific, South Pacific, and Indian Oceans, which can support the TC rapid intensification leading to increase the RI events^53^ (as seen in Fig. S6d).

**Figure 9:**
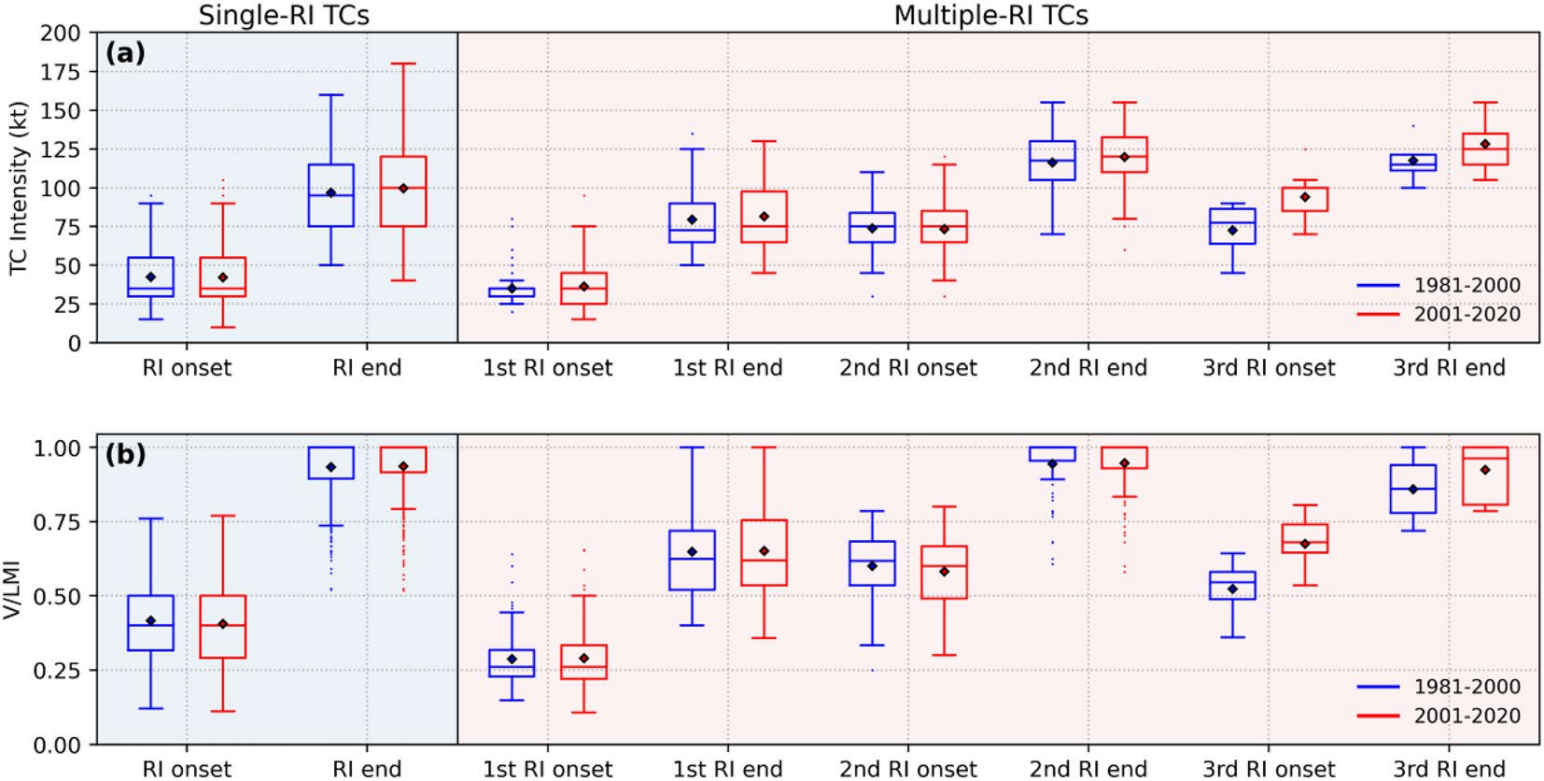
Distribution of the RI onset and RI end positions in TC life cycle in terms of (**a**) TC intensity and (**b**) V/LMI for single-RI TCs (shown in blue color background) and multiple-RI TCs (shown in red color background) between the periods P1 (blue) and P2 (red)**.** The rhombus symbol inside the box indicates the mean value of the distribution.

**Figure 10:**
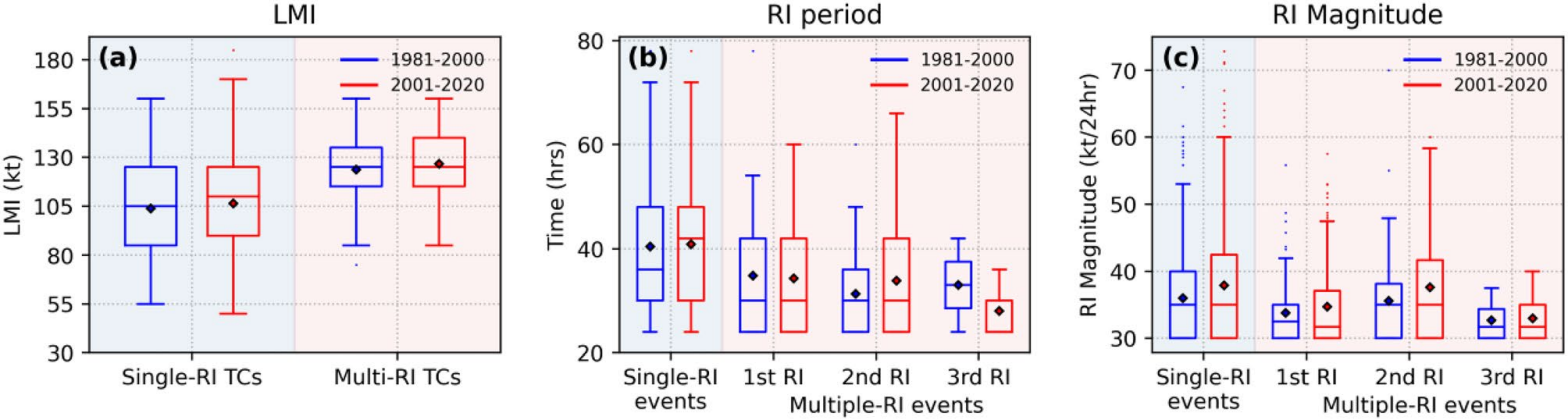
Box plots showing the variations in the distribution of (**a**) LMI (**b**) RI period and (**c**) RI magnitude of single-RI TCs and multiple-RI TCs between the periods P1 (blue) and P2 (red)**.** The rhombus symbol inside the box indicates the mean value of the distribution.

It is crucial to analyze the distribution of multiple-RI events within the life cycle of TC to understand multiple-RI TCs better. We have calculated the RI onset and RI end positions in the TC life cycle of both single-RI TCs and multiple-RI TCs in terms of TC intensity (Fig. 9a) and V/LMI (Fig. 9b). In scenarios characterized by conducive environmental conditions, the commencement of RI generally occurs during the TS stage. It persists until the TC intensity approaches its LMI, continuously progressing without interruptions. Consequently, these TCs evolve into single-RI TCs. As a result, single-RI events exhibit extended periods of the RI (illustrated in Fig. 10b). In the context of multiple-RI TCs, the initial RI event occurs at an average intensity lower (approximately 5.75 knots) than the TC's initial intensity of RI phase observed in singular-RI TCs. However, because of unfavourable environmental factors, this RI phase may end prematurely, causing the TC to either undergo a period of slow intensification or a slight weakening. Despite the interruption in the intensification process, under circumstances of high MPI, the intensity stage at which the first RI event concludes remains conducive for further RI. This scenario can lead to a second RI event in TCs if the environmental conditions are supportive. This second RI event may continue until the TC intensity reaches its LMI, or the same process may recur a few more times, increasing the number of multiple-RI events. With ongoing global warming leading to rising SSTs (also MPI) the number of RI events within the TC life cycle is anticipated to increase. As the number of RI events grows during the TC life cycle, it becomes noticeably more efficient in forcing the TC to reach higher LMI. Consequently, the average LMI for multiple-RI TCs is approximately 20 knots higher than that of single-RI TCs (Fig. 10a). In a warming scenario, the growing magnitude of RI events in TCs leads to higher LMI^52^, with Single-RI TCs experiencing an LMI increase of 0.20 knots/year (p = 0.01), and multiple-RI TCs exhibiting an LMI increase of 0.17 knots/year (p = 0.12) (see Fig. S2).

We conducted additional analyses using the RI thresholds 35 kt^18^ and 45 kt^51^. We found that the trends in multiple-RI events were still detectable and significant when using a 35 kt threshold (see Fig. S3). However, it became clear that these patterns were less pronounced when compared to the results obtained using the 30 kt threshold. Furthermore, we extended our analysis to include a 45 kt threshold (Fig. S4). In this case, we found that the trends in multiple-RI events were less apparent. The varying magnitude of the multiple-RI event within the TC life cycle is a crucial factor influencing this phenomenon. We observed that the RI magnitude tends to decrease as RI events occur closer to the MPI of the tropical cyclone. As the RI threshold is increased, it necessitates larger intensity changes for an event to be categorized as RI.

Consequently, when the threshold is set at 45 kt, it primarily captures second RI events with high magnitude and neglects initial and later RI events of the second RI event which were considered as RIs as per 30/35kt thresholds (Fig. 10c). This leads to fewer RI events satisfying the higher threshold, making the trends less apparent. In some instances, RI events with relatively small magnitudes occurring after a weakening in the TC's life cycle could lead to substantial prediction errors. These observations underline the importance of categorizing RI events based on their magnitudes rather than sticking to a fixed threshold. Figure 10c shows the differences between the magnitudes of single-RI and multiple-RI events during the two temporal regimes we selected. We know that the RI increases with the intensity of TC until it reaches close to the MPI. Single-RI events typically persists for a long time (Fig. 10b) without weakening, and their magnitude is usually significant. Since most of the 2nd RI events have occurred in post-TS stages, compared to the stages of the 1st RI occurrence (TD, TS stages), the mean magnitude is higher for the former. The occurrence of the 3rd RI being close to the MPI might have reduced its magnitude. The mean magnitude of the 1st, 2nd and 3rd RI of multiple-RI TCs has increased by 1 kt, 2 kt and 0.26 kt, respectively, while the mean magnitude of single-RI events has risen by 1.90 kt. RI duration for single-RI events shows a significant increase (0.082 h/year, p = 0.029), and the main RI event (second RI) of multiple-RI events also experiences a notable rise (0.11 h/year, p = 0.031). Rising MPI enhances post-TS stages for RI initiation or progression, correlating with extended RI periods in the TC life cycle. This explains the RI’s increasing magnitude and duration in response to increasing MPI due to global warming.

## Discussion and Summary

The primary goal of this study is to determine the trends of multiple-RI events of TCs by analyzing the variation in the stage-wise distribution of RI events. Historically, most RI TCs underwent RI during the initial phase (TS stage) of the TC life cycle. In our analysis of IBTrACS data for worldwide TCs developed between 1981 and 2020, we discovered that the chance of TCs undergoing RI has increased in the Cat-1,2,3 stages during the last 20 years. This shift in RI favorability to post-TS stages of TC increased the number of RI events within a TC life cycle, enhancing the frequency of multiple-RI TCs. The intensification rate of TC depends on its initial intensity^27^. The maximum intensification rate in a TC life cycle occurs at a TC intensity that should be moderate compared to the MPI (theoretical upper limit of LMI). Kaplan and Demaria^7^ also noticed that the chance of TC undergoing RI will be high when the TC intensity is significantly below its MPI. As the oceans warm, the MPI rises, and the TC intensity, where it experiences a maximum intensification rate, also increases. Thus, the post-TS stages of TC (cat-1,2,3) are more suitable for RI initiation or progression, as they are still far enough from the theoretical upper limit of TC intensity. However, external unfavorable factors can prematurely halt an initial RI phase, causing the TC to undergo slow intensification or slight weakening. Despite this interruption, in conditions of high MPI, the intensity stage where the first RI event concludes remains conducive to further RI. This can lead to a second RI event if environmental conditions support it. The second RI event may continue until the TC intensity reaches its Lifetime Maximum Intensity, or this process may recur multiple times, resulting in an increased occurrence of multiple-RI events. This process has raised the frequency of multiple-RI TCs by 82.43% in the last 20 years, while the frequency of single-RI TCs has increased by only 1.63%. A significant increase in frequency was observed across all ocean basins for both multiple-RI TCs and events.

TCs experiencing multiple-RI events result in higher Lifetime Maximum Intensity compared to single-RI TCs. Single-RI events often extend over longer uninterrupted periods, contributing to their higher magnitudes. Since the second RIs of multiple-RI TCs happen at TC intensities that are significantly higher but still sufficiently below MPI, they survive longer than other RIs in the life cycle and hence exhibit higher magnitudes. The RI period of single-RI events and the second RI of multiple-RI events is increasing significantly (Fig. S2) raising their mean magnitude by 1.9 kt and 2kt, respectively (Fig. 10c). Higher MPI can lead to more severe storms with an unpredictable nature by increasing multiple-RI events in the future. Further research is necessary to comprehend the full potential effects of MPI increase on TC intensification rates and develop strategies to reduce the risks of severe storms.

## Methods

In this study, we used the best-track data from IBTrACS version 4^45,46^ for global TCs that formed between 1981 and 2020. To ensure data consistency, best-track TC data from the National Hurricane Center was used for the North Atlantic (NA) and eastern-north Pacific (ENP) basins, while data from the Joint Typhoon Warning Center was used for the remaining ocean basins. Considering the differences in the long-term RI trend between different best track datasets, we cross-checked our main conclusion with additional best-track datasets, particularly those provided by WMO agencies available in IBTrACS. We have considered the data of TCs with LMI > 34 kt, located over water within 40°S − 40°N. The TC record of the standard observing times of 00, 06, 12, and 18 UTC has been kept for analysis. There are 3480 TCs selected in this study. 24-h intensity changes (ΔV_24_) are calculated as the difference in V_max_ of TC over every 24-h period. Here we treat positive ΔV_24_ as Intensification Rate (IR), and negative ΔV_24_ as a weakening rate (WR), further, it is considered a neutral Intensification Rate (NIR) if there is no change in the intensity over a 24-h period (ΔV_24_ = 0 kt). The study period is divided into two equal halves, 1981–2000: 2001–2020 (P1, P2 henceforth). To check the relative variations of different ΔV_24_ within each stage of TC, IRs are further classified into rapid intensification rates (RI rates; where 24-h IRs ≥ 30 kt) ^7^ and non-RI rates (non-RI; where 24-h IRs = 1 kt to < 30 kt). We employed the approach introduced by Kieper and Jiang^50^ to detect RI events in TCs. To quantify RI, we computed the ΔV_24_ as the difference in V_max_ of TC over every 24-h period. This calculation began from the TC's initial 6-h data point, and subsequently, a ΔV_24_ value was determined for every 6-h data point. Each 24-h period with an intensity variation ≥ 30 kt is defined as a RI period. Later all consecutive RI periods (overlapping), were then grouped together into a RI event. To put it in other way, a RI event has at least one RI period that lasts for 24 h, whereas a prolonged RI event has numerous (overlapping) RI periods and might last for several days. The mean magnitude of all RI periods in a RI event is considered as RI magnitude. Here, we define single-RI TCs as those who have undergone RI only once in their lifetime. A TC may be subjected to RI multiple times during their lifetime. Following the initial RI, TC may continue to intensify at a non-RI rate or may weaken before next RI event. A TC is considered multiple-RI if it features more than one RI event separated by a non-RI /negative 24-h intensity change. There are no overlapping periods among the multiple-RI events. Through this procedure, a total of 1322 RI-TCs were identified, out of which 1113 were classified as single-RI TCs and 209 were categorized as multi-RI TCs. All the atmospheric and oceanic parameters used in the present analysis are obtained from the European Centre for Medium-Range Weather Forecasts' fifth-generation reanalysis (ERA5)^47^. MPI calculations were done using Gilford’s pyPI algorithm^48^, which is an application of the Bister and Emanuel algorithm^49^. We calculated the statistical significance of trends of time series using linear rgression, differences in mean ΔV_24_ between two study periods using the ANOVA test and changes in the distribution of initial intensity where TC undergone maximum 24-h intensity change using Kolmogorov–Smirnov test. The differences in the probabilities of 24-h intensity changes between different categories (Fig. 3) is determined using a z-test for proportions.

### Supplementary Information

Supplementary Information.

## Data Availability

Tropical cyclone data is publicly available from the International Best Track Archive for Climate Stewardship (IBTrACS). (https://www.ncei.noaa.gov/data/international-best-track-archive-for-climate-stewardship-ibtracs/v04r00/access/netcdf/). All atmospheric and oceanic parameters were obtained from the European Centre for Medium Range Weather Forecasts Reanalysis 5 (https://www.ecmwf.int/en/forecasts/datasets/reanalysis-datasets/era5).
