# Supplementary material for: Recent global increase in multiple rapid intensification of tropical cyclones
Source: Sci Rep. 2023 Sep 24;13:15949. doi: 10.1038/s41598-023-43290-9 (PMC10518314; doi:10.1038/s41598-023-43290-9)
Supplement: Supplementary file 1 — Supplementary Information. [file 41598_2023_43290_MOESM1_ESM.pdf]

Supplementary information for

Recent global increase in Multiple Rapid Intensification of Tropical Cyclones

N.D. Manikanta [1,2], Sudheer Joseph [1 *], C.V. Naidu [2]

[1] Indian National Centre for Ocean Information Services, Ministry of Earth Sciences, Hyderabad, India.
[2] Department of Meteorology and Oceanography, College of Science and Technology, Andhra University, Visakhapatnam, Andhra Pradesh, India.

Introduction

The supplementary information file comprises six figures and a table. Fig. S1 illustrates the sample tracks of both single-RI and multiple-RI tropical cyclones utilized in this study. Fig. S2 displays the trends in the occurrence of rapid intensification (RI) and the associated periods for both single-RI and multiple-RI tropical cyclones. Fig. S3 presents the trends and periodic variations in the frequency of single-RI and multiple-RI tropical cyclone events when considering an RI threshold of 35 knots. Fig. S4 presents a similar analysis to Fig. S3 but with a 45-knot RI threshold. Fig. S5 extends the analysis from Fig. S4 to a subset of data from WMO agencies within the IBTrACS dataset, using a 30-knot RI threshold. Finally, Fig. S6 depicts the changes in the environmental conditions between the two study periods. Tabe S1 gives the details about the the differences between the mean 24-hour intensity changes during different intensity stages of TCs for global TCs, RI-TCs and non-RI TCs corresponding the Fig. 1, 2 in the main manuscript.

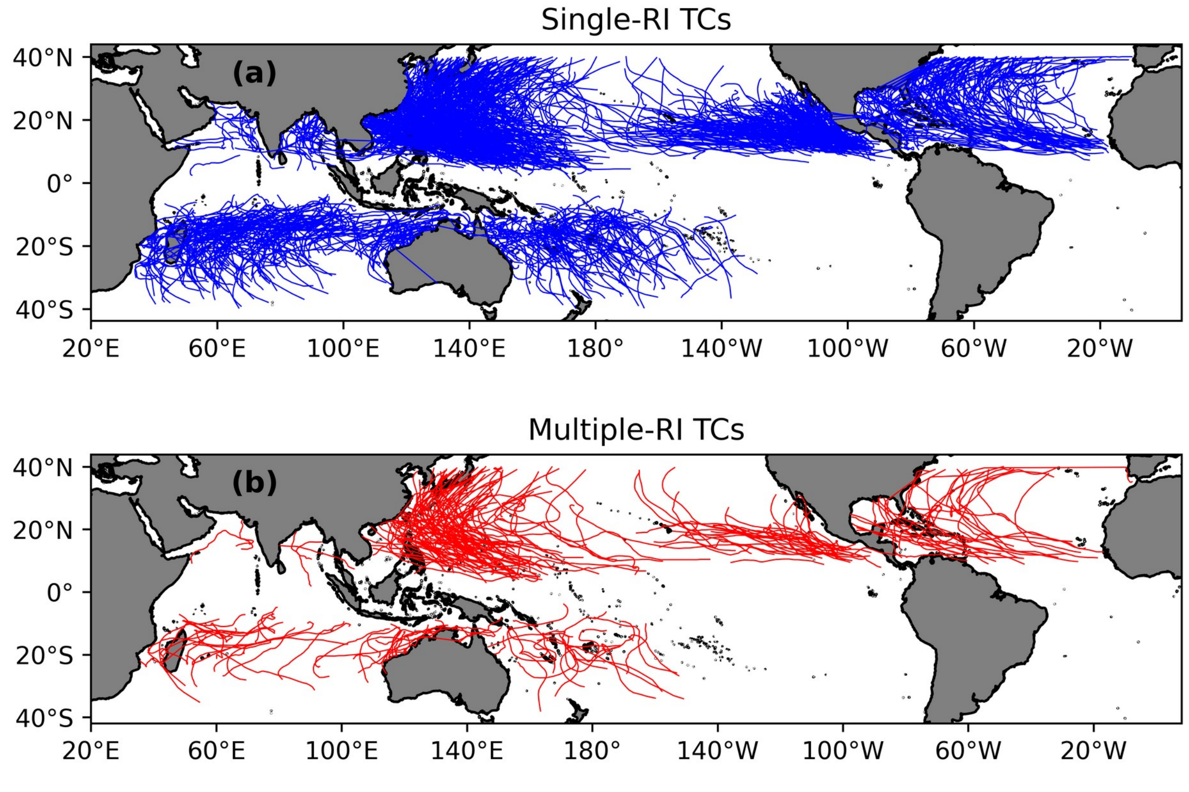

Figure S1 showing the tracks of all (a) single-RI TCs and (b) multi-RI TCs used in the present analysis . Tracks are based on TC records from Joint Typhoon Warning Center (JTWC), National

Hurricane Center (NHC) in the IBTrACS dataset. The Matplotlib (https://pypi.org/project/matplotlib/) (version-3.7.1) and Cartopy (https://pypi.org/project/Cartopy/) (version-0.21.1) modules of python was used to create the figure.

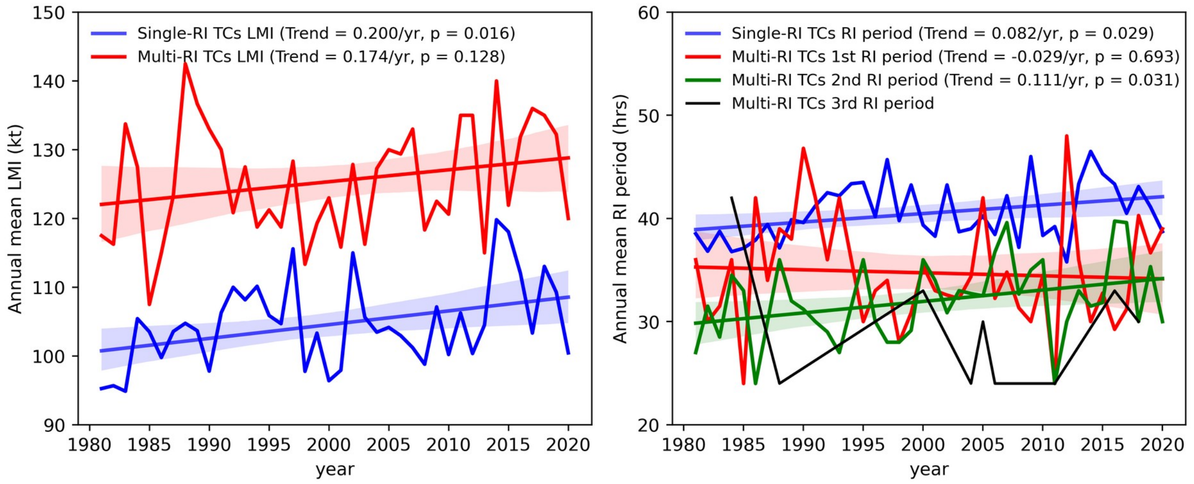

Figure S2 showing the trends of (a) LMI of single-RI TCs and multiple-RI and (b) RI period of single-RI TCs and multiple-RI TCs. LMI and RI period trends are based on TC records from Joint Typhoon Warning Center (JTWC), National Hurricane Center (NHC) in the IBTrACS dataset. The trends and p-values this figure are calculated using linear regression analysis. The trends and p-values this figure are calculated using linear regression analysis. The Matplotlib module (version-3.7.1) of python (https://pypi.org/project/matplotlib/) was used to create the figure.

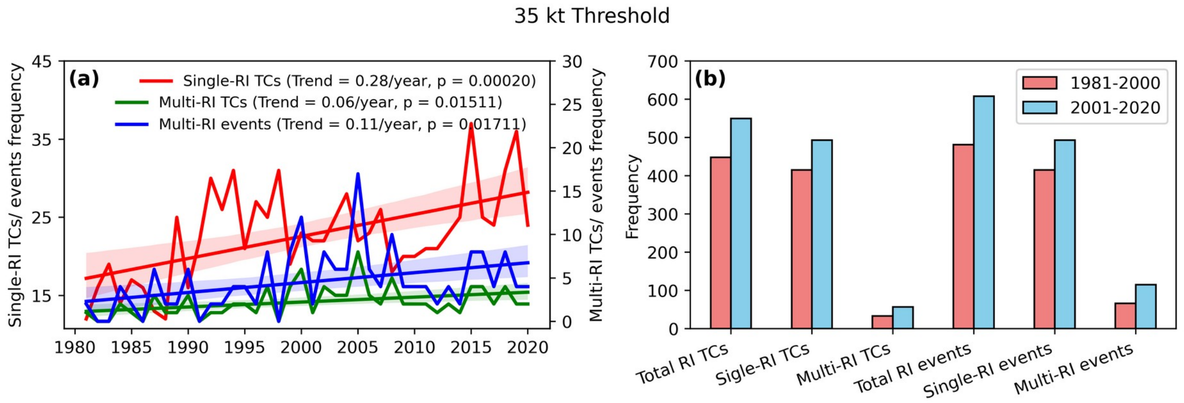

Figure S3: Time series and trend of annual frequency for single-RI TCs (in red), multiple-RI TCs (in green), and multiple-RI events (in blue) at the 35 kt threshold during the period 1981–2020. (b) Frequency changes for total RI-TCs and total RI events, single-RI TCs and events, and multiple-RI TCs and events between the periods P1 and P2. Multi-RI and single-RI trends for 35 knot threshold are based on the IBTrACS dataset. The trends and p-values this figure are calculated using linear regression analysis. The Matplotlib module (version-3.7.1) of python (https://pypi.org/project/matplotlib/) was used to create the figure.

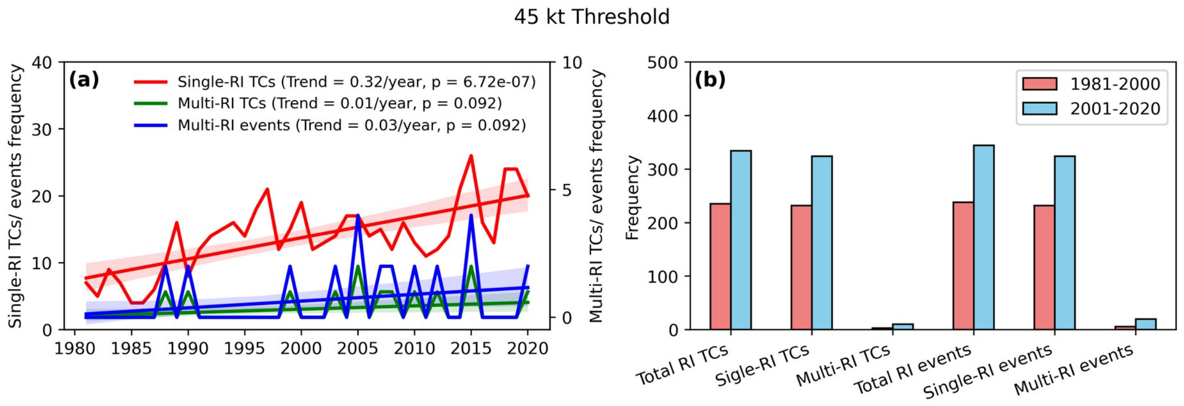

Figure S4: Time series and trend of annual frequency for single-RI TCs (in red), multiple-RI TCs (in green), and multiple-RI events (in blue) at the 45 kt threshold during the period 1981–2020. (b) Frequency changes for total RI-TCs and total RI events, single-RI TCs and events, and multiple-RI TCs and events between the periods P1 and P2. Multi-RI and single-RI trends for 45 knot threshold are based on the IBTrACS dataset. The trends and p-values this figure are calculated using linear regression analysis. The Matplotlib module (version-3.7.1) of python (https://pypi.org/project/matplotlib/) was used to create the figure.

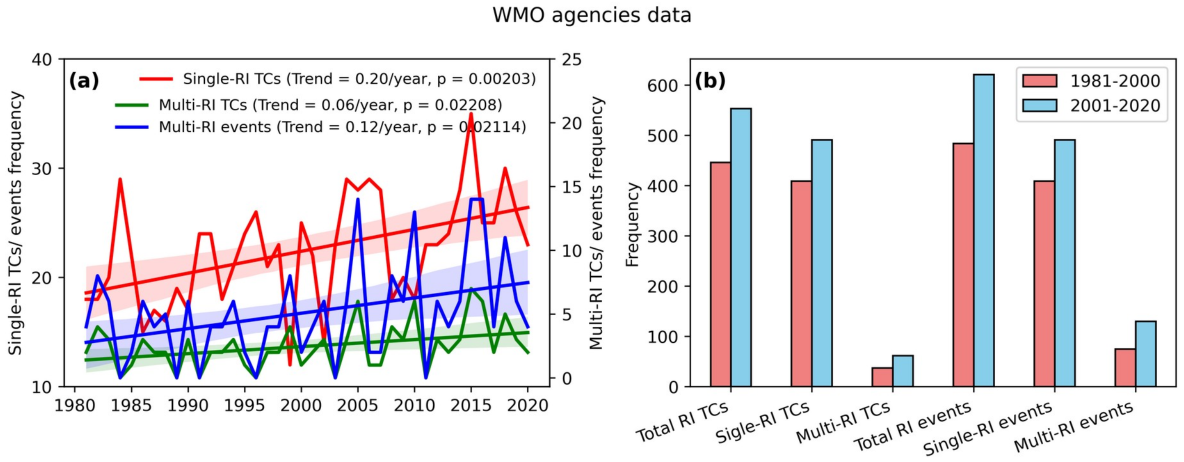

Figure S5: Time series and trend of annual frequency for single-RI TCs (in red), multiple-RI TCs (in green), and multiple-RI events (in blue) for WMO agencies data during the period 1981–2020. (b) Frequency changes for total RI-TCs and total RI events, single-RI TCs and events, and multiple-RI TCs and events between the periods P1 and P2. Multi-RI and single-RI trends are based on the WMO agencies dataset in IBTrACS. The trends and p-values this figure are calculated using linear regression analysis. The Matplotlib module (version-3.7.1) of python (https://pypi.org/project/matplotlib/)was used to create the figure.

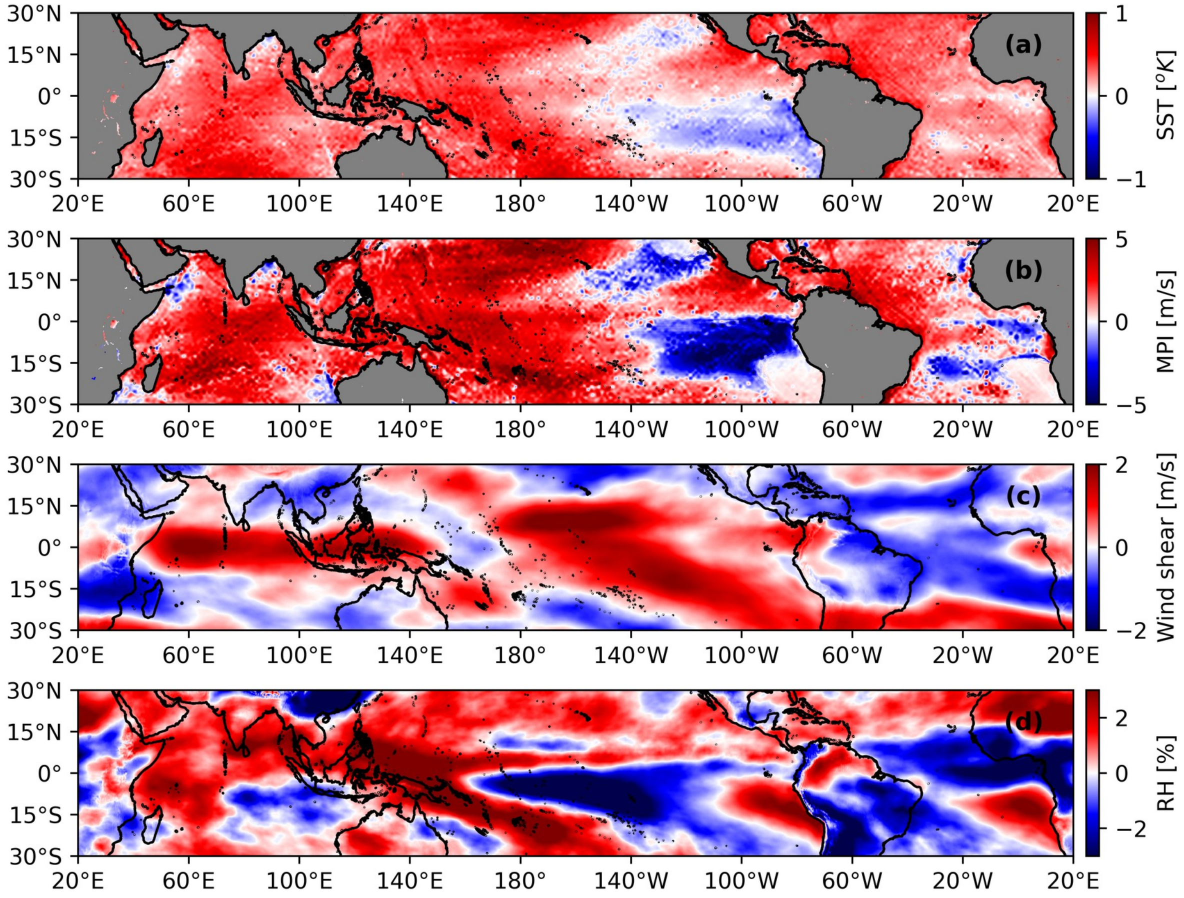

Figure S6: Maps depicting variations of environmental conditions between two study periods a) Sea surface temperature (K), (b) Maximum potential intensity (m/s), (c) 200–850 hPa vertical wind shear (m/s), and (d) 600 hPa relative humidity (%). All the environmental parameters in the above map are based on the ERA5 dataset. The Matplotlib (https://pypi.org/project/matplotlib/) (version-3.7.1) and Cartopy (https://pypi.org/project/Cartopy/) (version-0.21.1) modules of python was used to create the figure.

	Global TCs	RI -TCs	Non-RI TCs
TD	2.28	3.004	1.742
TS	0.92	1.056	-0.53
Cat - 1	5.029	5.451	-0.06
Cat - 2	4.149	3.004	2.537
Cat - 3	0.29	-0.0026	0.477
Cat - 4	0.328	0.84	-6.26
Cat - 5	-0.234	-0.106	--

Table S1- Differences in the mean 24-hr intensity changes during different intensity stages of TCs corresponding to Figure 1 and Figure 2.
